# Introducing patient perspective in health technology assessment at the local level

**DOI:** 10.1186/1472-6963-9-54

**Published:** 2009-03-27

**Authors:** Marie-Pierre Gagnon, Dolorès Lepage-Savary, Johanne Gagnon, Michèle St-Pierre, Chantale Simard, Marc Rhainds, Renald Lemieux, François-Pierre Gauvin, Marie Desmartis, France Légaré

**Affiliations:** 1 Quebec University Hospital Research Centre, Quebec, Canada; 2 Department of Nursing, Laval University, Quebec, Canada; 3 Quebec University Hospital Centre, Quebec, Canada; 4 Department of Management, Laval University, Quebec, Canada; 5 Sherbrooke University Medical Centre, Sherbrooke, Canada; 6 Department of Family Medicine, Laval University, Quebec, Canada

## Abstract

**Background:**

Recognizing the importance of increased patient participation in healthcare decisions leads decision makers to consider effective ways to incorporate patient perspectives in Health Technology Assessment (HTA) processes. The implementation of local health HTA units in university hospitals in Quebec provides a unique opportunity to foster an increased participation of patients in decisions regarding health technologies and clinical interventions. This project explores strategies that could be effective in involving patients in HTA activities at the local level. To do so, three objectives are pursued: 1) To synthesise international knowledge and experiences on patient and public involvement in HTA activities; 2) To explore the perceptions of stakeholders (administrators, clinical managers, healthcare professionals, HTA producers, and patients) regarding strategies for involving patients in various HTA activities; and 3) To produce a consensual strategic framework that could guide interventions for involving patients in HTA activities at the local level.

**Methods:**

A systematic review of the literature will be conducted to synthesise international knowledge and experiments regarding the implication of patients and public in HTA. Then, focus groups will be carried out with representatives of various stakeholder groups in order to explore their perceptions regarding patient participation in HTA. Based on findings from the systematic review and the focus groups, a framework to support patient participation in HTA activities will be proposed. It will then be validated during a deliberative meeting with the research team, composed of scientists and decision makers, and representatives from different groups involved in HTA in Quebec. This deliberative meeting will aim at identifying the type and the degree of participation as well as the adequate timing for involving patients in local HTA activities.

**Discussion:**

Given the actual state of evidence, integrating patient perspective in HTA activities has the potential to improve the quality of healthcare services. This study provides an opportunity to bridge the gap between HTA producers and its ultimate end-user: the patient. It will provide guidance to support local HTA units in Quebec and elsewhere in their decisions regarding patient participation. The framework developed could be applied to design and implement strategies for involving patients in HTA activities.

## Background

Improving the quality of healthcare through the best scientific evidence available in a context of scarce resources is a challenge for health systems [1]. Health technology assessment (HTA) is a multidisciplinary field of applied research aimed at providing high-quality information about the clinical effectiveness, cost-effectiveness, and broader impact of drugs, medical technologies, and health interventions in order to support and inform those who make decisions about health policy and purchasing, health services organisation and management, and clinical practices [2,3]. In Canada, the Canadian Agency for Drugs and Technologies in Health (CADTH), and provincial agencies, such as the Agence d'évaluation des technologies et des modes d'intervention en santé (AETMIS) in Quebec, are responsible for conducting health technology assessments at a macroscopic level. However, HTA recommendations are seldom transferred into decisions and practices [4]. In order to favour knowledge transfer and to support evidence-based decision making at both mesoscopic and microscopic levels, local HTA units have been implemented in all five university hospitals in Quebec. The implementation of these local HTA units is viewed as a strategy to improve the relevance and timeliness of HTA recommendations and, ultimately, to facilitate their uptake [5]. This implementation could thus favour a culture of evaluation in university hospitals, which is coherent with the promotion and uptake of effective healthcare decisions.

Effective healthcare decisions are more and more conceptualised as the best course of action given the current scientific evidence, healthcare resources, clinical circumstances, and patient preferences [6]. With the increased emphasis on the engagement of patients as full partners in their care, there is a need to determine effective ways to involve them in the decision process [7,8]. It is expected that the need for "*patient guidance will only increase as clinical options multiply and the world of information continues its rapid growth*" [9]. This is congruent with the fact that several HTA agencies and academics associated with HTA are now considering ways to incorporate the perspectives of patients in their methods, thus calling for patient-centred HTA [10,11].

The literature proposes different terms to refer to the various groups of end-users of HTA, such as patients, consumers, citizens and public. There is a lack of terminological consensus with the term public involvement. The analysis by Gauvin [12] identified six publics that he grouped into two categories. Publics "*whose role is to provide a societal or lay perspective about health technologies*" constitute the first category that includes citizens, groups representing citizens, and other representatives such as elected officials. The second category consists of "*those publics directly affected by a given health condition or health technology*" and includes individual patients, services users, and the entities representing them [12]. According to Cayton [13], these two categories could be considered as the two sides of the same coin because they represent different roles individuals could take when they engage with health services. However, according to Gauvin [12], most experts in HTA believe that the broader perspective of citizen is more likely to be involved in the organizational and policy-making domains and the perspective of patients and service users in the research domain. In this last domain, patients and service users are better positioned to highlight matters relevant to service users such as social and ethical consideration, patient acceptance of the technology, and psychological implications. In this study, we are interested mainly by patients because they are the group most directly affected by HTA decisions, and their unique insight can most usefully contribute to the HTA process. They could provide 'experiential' evidence to the HTA process [14]. We include members of the public (*i.e *citizens) in our literature review because of their role in some HTA activities and given the lack of terminology consensus about these terms. However, the other stages of the project are focusing more precisely on the patients.

The value of an increased participation of patients in healthcare decisions is now recognised and leads decision makers to consider effective ways or strategies to incorporate patients' perspectives in decision making processes.

### Gaps in knowledge this study is addressing

Although opportunities to favour patient participation in HTA activities are increasingly considered [11,15], few experiences of patient involvement in HTA appear to be reported in the literature [16]. Furthermore, these experiences mostly concern national HTA agencies, such as NICE and NCCHTA in England [17,18] and DACEHTA in Denmark [19]. Also, few studies have examined the participation of patients, consumers, or the general public in priority setting regarding health technologies [20-22]. To the best of our knowledge, patient involvement in university hospital-based HTA units has not been studied yet. According to a survey among Canadian health consumer groups representing various diseases or conditions, respondents reported a desire for greater involvement in HTA, and provided feedback on mechanisms for facilitating their participation [23]. The HTA program of the Alberta Heritage Foundation for Medical Research in Canada has identified a need for greater public involvement in its strategic planning, particularly for the identification of potential topics of investigation [24]. Other countries, such as England [18] and Denmark [19], have also suggested increased citizen and patient participation in HTA.

However, several gaps in knowledge remain in order to support the implication of patients in HTA. These gaps concern, for instance, the willingness of patients and members of the public to be involved in healthcare decisions, including the nature of their involvement [25], the attitude of healthcare professionals regarding patient involvement [26-28] and the absence of proven effective strategies to guide their implication [29,30].

### Goal, objectives and conceptual framework

The aim of this project is to explore how the patient perspective could be introduced into the structures and activities of a local HTA unit. To do so, three objectives are proposed: 1 – To synthesise international knowledge and experiences on patient and public involvement in HTA activities; 2 – To explore the perceptions of stakeholders (administrators, clinical managers, health professionals, HTA producers, and patients) regarding patient participation in the various HTA activities, effective strategies to support their participation, as well as barriers and facilitators to this participation; and 3 – To produce a consensual framework to guide interventions supporting patient participation in HTA activities at the local level. The research will use analytical frameworks developed in the field of patient involvement in clinical decision making [31,32] and public participation in research and HTA activities [11,12,23,33] for each objective of the research.

## Methods/Design

Two local HTA units participate in this study. One is located at the Quebec City University Medical Centre and the other one, at the Sherbrooke University Medical Centre. The implementation of these two local HTA units provides a unique opportunity to assess the feasibility of introducing patient perspectives in hospital HTA activities.

Globally, we adopt a pluralist action research strategy [34] that will allow constant adjustments between research objectives, methods, data collection, and analysis. This strategy seems the most appropriate, given the specific nature of the partnership between researchers and decision makers in this project. With regards to utility for decision making, this collaboration between researchers and decision makers is fundamental. The role of decision makers is to ensure the appropriateness of the research questions, objectives, methods, analysis, and outcomes to the context, and the relevance of the research for its potential beneficiaries. Researchers have a role of facilitators in order to translate decision makers' needs into a systematic and rigorous research process and to analyse factors influencing the decision-making behaviours.

### Phase one: Systematic review of international experiences of involving patients in HTA

For Objective 1, a systematic review of the scientific literature (qualitative, quantitative and mixed-methods studies) and other published documentation (technical or grey literature) will be conducted to document international experiences regarding patient involvement in HTA. It will also consider the concept of public involvement that is broader and allows the inclusion of various levels, types and contexts of involvement of different types of public. This review will synthesise knowledge on the strategies and activities regarding patient and public involvement in HTA, and the potential impact of this involvement, notably on clinical interventions, costs, timeframes, and perceptions of other stakeholder groups.

Previous reviews and syntheses by researchers of the team [26,35-39] will guide the elaboration of the search strategies. Standardised literature searches will be conducted in all relevant databases (PubMed, Embase, CINAHL, PsycINFO, Cochrane Library, Science Citation Abstract, Social Science Citation Asbtract, Business Source Premier, ABI/Inform, Dissertation Abstract) and in the International Journal of Technology Assessment in Health Care (IJTAHC). Relevant references from studies found through the above sources will be followed up and obtained for assessment. Other literature will be identified through Internet search engines and governments' websites. Finally, publications citing the selected articles as well as other articles by authors of the selected articles will be searched through the ISI Science Citation Index.

The sensibility of the search strategy will be validated by ensuring that all relevant articles identified by team members (including decision makers and researchers) are retrieved. Experts in the field of HTA will be contacted for unpublished studies. The diversity of interests and expertise among team researchers and their respective networks will ensure that all relevant literature is covered.

After a first selection of potentially relevant articles, full text copies of these papers will be retrieved and screened independently by one of the two principal investigators (MPG and FL) and a research assistant to assess which studies fit the inclusion criteria. Then, each study will be independently abstracted by teams of two reviewers among the team researchers.

Studies will be abstracted and appraised using consensual guidelines for narrative syntheses and meta-analytical techniques [40-43]. Findings will be reported using frameworks that propose models of patient and public involvement in HTA [11,23,33]. The main characteristics that will be extracted include: the type and the level of involvement as well as the timing. Other elements, such as effects on participants and on decisions about HTA, as well as factors facilitating or limiting patient participation will also be considered.

### Phase two: Focus groups on patient participation in HTA

For Objective 2, focus groups will be conducted among representatives of the various groups of stakeholders involved in the project (hospital administrators, clinical managers, healthcare professionals, HTA producers, and patients) to explore their perceptions regarding patient participation in HTA. A semi-structured guide will be used to explore participants' perceptions about the importance and relevance of each model of patient participation found in the literature, as well as the applicability of the model to the specific context of hospital-based HTA. Focus groups will also explore participants' knowledge, beliefs, and attitudes toward patient participation in HTA, and specific questions will be asked about the desired type of patient participation given the specific technology or clinical intervention considered. A total of five focus groups are expected with a number of participants varying from 5 to 8, as recommended by experts [44,45]. Potential participants will be identified through the contact network method, which consists of contacting a representative of each group concerned and asking him or her to identify potential participants [46] who will be invited to take part in the project. Informed consent will be sought from all participants. The focus groups will last approximately two hours and will be recorded, with the consent of participants.

All information obtained from focus groups will be transcribed *verbatim *and qualitative analyses will be performed with N*Vivo. The method proposed by Huberman & Miles [47] will guide the analyses. This method identifies three steps in qualitative data analysis: coding, organisation and correlation of data. Qualitative data will be coded according to predefined variables from the analytical frameworks used, but will also allow the identification of emerging themes. During the initial stages of the analysis, two of the researchers will independently code the transcripts, and codification will be compared between them in order to enhance the reliability of the coding. In case of divergence, a third researcher will be consulted and a consensus will be obtained through discussion. Furthermore, three representatives from each study group will be invited to comment a first draft of the report so that their interpretation of findings and their suggestions for recommendations could be incorporated.

### Phase three: A consensual framework to guide intervention

For Objective 3, a preliminary report presenting the key results of the systematic review and the focus groups, including a framework of reference to support patient participation in HTA activities, will be presented and discussed during a deliberative meeting. This meeting will bring together all members of the research team, researchers and decision makers collaborating to the project, representatives of other HTA units across the province of Quebec, and patient representatives. Between 8 and 10 participants are expected to attend the meeting. As proposed by Pagliari et al. [48], the three stages of group decision making will be followed: 1) definition of the problem; 2) discussion of possible alternatives; 3) decision. The results of the systematic review and the focus groups will be first presented and a strategic framework for patient participation in HTA will be proposed. Participants will be invited to share their opinions about the relevance and applicability of the proposed models and strategies to support patient participation in HTA. This deliberative meeting will allow direct interaction between researchers and stakeholders about models found in the literature and the relevance of the proposed framework to their reality. This meeting will also allow the clarification of the major points of consensus and disagreement in order to estimate the robustness and the transferability of the results [41,49], as well as the questions that require more extensive research [50].

Following the deliberative meeting, the proposed strategic framework will be enhanced by combining knowledge about models of patient participation found in the literature, specific characteristics of the context from the focus groups and the deliberative meeting. The core components of this strategic framework will be the type of participation, the degree of involvement, and the timing. This framework will be used in the development of strategies aiming at the participation of patients in specific HTA activities and will guide the evaluation of outcomes in the following phase of this research program. This framework could easily be transferred to other HTA institutions in Quebec and outside the province. This framework could also constitute a useful guide to support decision making regarding the implication of patients in other domains, such as prioritisation in risk management strategies or policies on the management and control of infections.

## Knowledge translation (KT) plan

This project aims at producing usable knowledge that could support decision makers responsible for HTA activities. According to a systematic review of factors determining knowledge application in decision making, interactions between researchers and healthcare policy makers and timing/timeliness are two factors that mostly appear to increase research use [51]. Our approach to knowledge translation is based on these two key-elements and is organised around three broad strategies.

The first strategy is the participation of stakeholders in each phase of the project. Notably, decision makers are involved in the elaboration of research objectives, the choice of the methodology, and the analysis and interpretation of results. To do so, workshops will be held with decision makers on the research team through a participatory approach [52]. These workshops will take place at key moments before and after each phase of the project. These transfer activities will allow the validation of research products according to decision makers' needs and comments. This strategy aims to ensure that the results are useful and usable to support decision making processes. The participation of decision makers in the formulation and implementation of research represents, according to Lomas [53], the best predictive element of the application of knowledge resulting from research.

The second strategy involves a representative of patients in HTA activities. This representative will participate in the meetings of the project team in order to validate the research processes. In addition, other representatives of patients will be solicited during the course of the project to take part in a focus group on their perceptions about patient participation in HTA activities. This direct participation of patient representatives will allow the consideration of their preferences in the proposed strategies. Moreover, the deliberative meeting will allow direct exchanges between hospital administrators, clinical managers, HTA producers and patients' representatives. This activity will allow the discussion of research findings in order to ensure that they are uniformly understood by all groups and are relevant to their decision-making needs. Following this deliberative meeting, consensual key messages will be elaborated for larger diffusion [54].

Finally, the third strategy is the transfer of research results to other HTA units in Quebec, and to other groups involved in HTA in Canada and elsewhere. This transfer will be done through participation of representatives from other HTA units of the province in the focus groups and the deliberative meeting. Collaborators from HTA agencies in other countries will also be informed about this initiative and their feedback will be sought through workshops organised at international HTA meetings.

At the end of the project, we will organise a provincial symposium targeting key decision makers (policy makers, managers and professionals) from the UETMIS, AETMIS and CADTH. This will be a unique occasion for all groups involved in HTA in the province of Quebec to present and share their research experiences with other teams. The symposium proceedings and presentations will be available on the Web through an open access. Decision makers from the Ministry of Health and regional health authorities will also be invited to this symposium.

### Evaluation of the knowledge translation plan

To evaluate the impact of the proposed KT plan, we will adapt a tool that was developed by Skinner [55] to measure knowledge exchange outcomes. This tool is based on a conceptual framework proposed by Knott & Wildavsky [56] and adapted by Landry et al. [57,58] The extent of knowledge application regarding patient participation in HTA activities will be measured based on a scale that will be developed in this study. The criteria to judge whether knowledge is adapted to the context and needs of stakeholders will be elaborated for each group. In order to ensure a formative evaluation of the different KT activities, a short questionnaire will be distributed to participants at the end of each activity. To measure the effective use of knowledge resulting from our research in decision making processes, we propose an approach based on the psychosocial determinants of human behaviours to evaluate the relevance of knowledge resulting from research from the perspective of the targeted audience, as well as their intention to use this knowledge to support their decision making. The questionnaire will require approximately 10 minutes to fill out. Its content will be analysed at the end of the project in order to measure the intention of decision makers to use the results in their practice.

## Ethical considerations

All data collected for the document analysis in this study will be obtained from publicly available sources. Participants in the focus groups (phase 2) will be asked to complete a consent form presenting research objectives and information about research implications. Ethics approval for the project has been received from the Research Ethics Board of the Centre Hospitalier Universitaire de Québec (approved December 18 2008; ethics number 5-08-12-05).

## Discussion

This study will be the first, to the best of our knowledge, to systematically review international experiences on patient participation in HTA, and to propose a framework that will support the development of a guide to support local HTA units in their decisions regarding patient participation. It will also explore the perceptions of various HTA stakeholders regarding the implication of patients in healthcare decisions.

This study is being conducted in close collaboration with decision makers in order to allow constant linkages that are likely to favour the relevance and utilisation of research results. Based on results from the literature review, focus groups, and deliberative meeting, effective strategies for supporting patient participation in HTA will be identified. A subsequent phase of this project could be the implementation of such strategies in the local HTA unit of university hospitals of Quebec. Thus, this research is an essential first step to ensure the feasibility of introducing patient participation in HTA.

Also, members of our research team are involved in two other knowledge syntheses that share a similar focus. One deals with patient perspectives on an electronic health record [59]. The other deals with patients and public involvement in clinical practice guidelines [60].

Given the actual state of evidence, promoting patient participation in HTA could improve the quality and safety of healthcare. This project provides an opportunity to bridge the gap between the producers of HTA and its ultimate end-user: the patient. The knowledge that will be produced will be extremely useful for policy makers, healthcare managers, and academics interested in HTA nationally and internationally, as well as for the larger community of people interested in the active participation of citizens and patients in healthcare decision making. This project directly aligns with at least three of the Canadian Institutes of Health Research strategic priority research areas: 1) it will provide innovative insight on how to successfully involving the public in decision making and, in particular, strategies for engaging the public in priority-setting; 2) it will reinforce a patient-centred care approach in focusing on the participation and involvement of patients; and 3) it will serve as a strategy to increase quality of care and patient safety [61].

## Competing interests

The authors declare that they have no competing interests.

## Authors' contributions

All authors collectively drafted the research protocol and approved the final manuscript. MPG is its guarantor.

## Pre-publication history

The pre-publication history for this paper can be accessed here:


